# Surgical Management of a Rare Complication of Mastoidectomy: A Retroauricular Cutaneous-Mastoid Fistula

**DOI:** 10.7759/cureus.84030

**Published:** 2025-05-13

**Authors:** Archil Tsuladze, Irine Nakhutsrishvili

**Affiliations:** 1 Otolaryngology, American Hospital Tbilisi, Tbilisi, GEO

**Keywords:** chronic mastoiditis, chronic suppurative otitis media, cutaneous-mastoid fistula, hearing loss, mastoidectomy

## Abstract

Our goal was to describe a case of a rare complication that developed as a result of multiple surgical interventions over the years due to chronic mastoiditis - specifically, a pathological connection between a retroauricular skin defect and the mastoid cavity - and its surgical management. Based on subjective and objective findings, a surgical intervention was planned, including radical cavity obliteration and closure of the cutaneous-mastoid fistula using the temporomastoid periosteum. The surgery was successfully completed, resulting in improved hearing and the elimination of the infectious focus.

## Introduction

The initial enthusiasm for surgical treatment methods for cholesteatoma and chronic mastoiditis soon evolved into a dogmatic controversy. Due to unfavorable experiences associated with inevitable recurrences, the concept of an individualized approach was developed over the years [1]. The treatment plan for mastoiditis depends on the patient’s toxicity and includes antibiotic therapy, tympanic membrane ventilation tube placement, and radical mastoidectomy [2,3]. In the literature, a postauricular fistula between the skin and mastoid is described as a rare complication following mastoidectomy, primarily caused by recurrent chronic suppurative otitis media or persistent infections. Bilateral manifestations of a cutaneous-mastoid fistula have also been reported as the ultimate complication of atticoantral chronic suppurative otitis media and acquired cholesteatoma [4,5]. The proper surgical management of such a fistula presents a significant challenge due to the presence of necrotic areas in the surrounding tissues [6].

## Case presentation

The patient, a 32-year-old woman, presented with complaints of left-sided hearing loss and purulent ear discharge persisting for several months. From the medical history, it was revealed that the patient had undergone four surgical interventions in the left ear over the past 18 years. The first surgery was performed at the age of 5 due to chronic suppurative otitis media, the second at the age of 10 for the same complaint, the third at approximately 13 years old, and the most recent at 22 years old. During the last procedure, a partial mastoidectomy was performed, and a foreign body - a bead - was found in the mastoid cavity.

**Figure 1:**
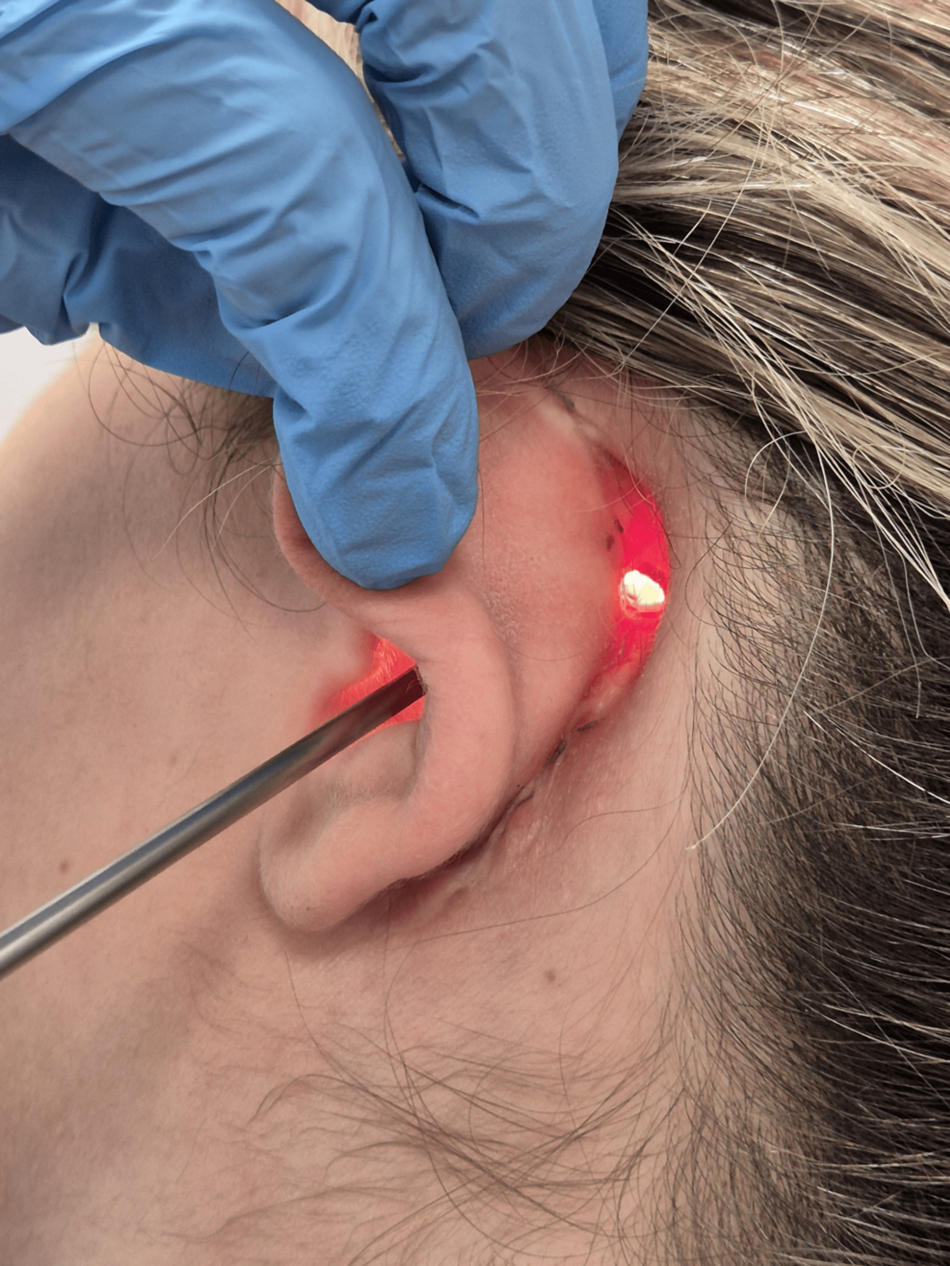
Retroauricular cutaneous-mastoid fistula The preoperative image describes the pathological connection between the skin and the mastoid.

**Figure 2:**
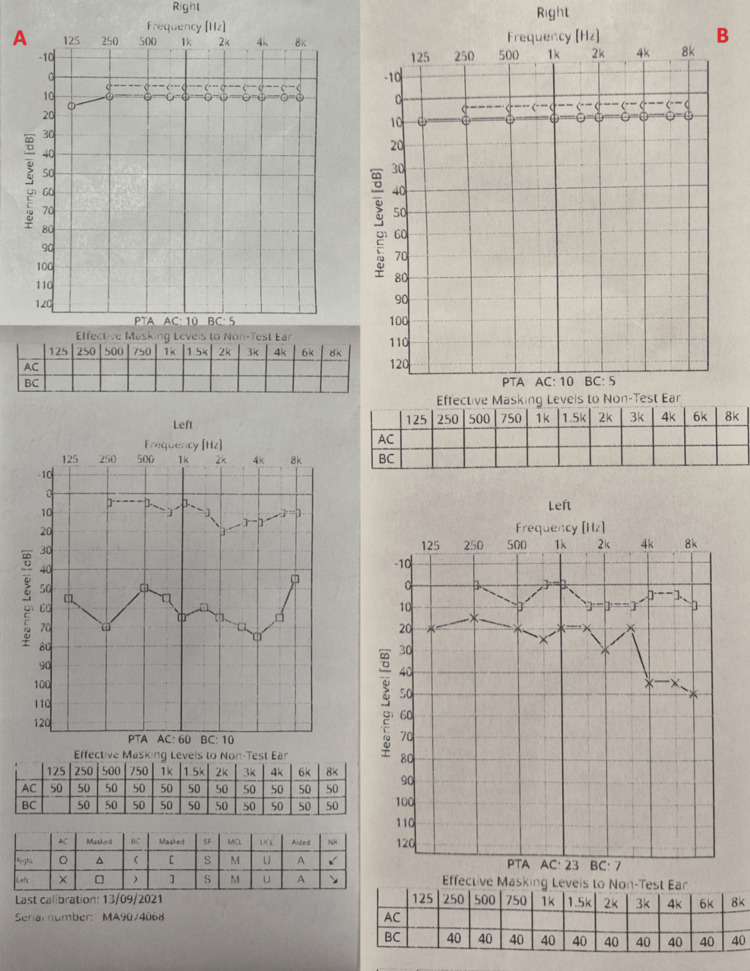
Pre- and postoperative audiometry, showing improvement in conductive hearing loss A: Preoperative audiometry showing left-sided conductive hearing loss of 60-70 dB; B: Postoperative audiometry: one month after the surgery, a hearing evaluation was performed through audiometry, revealing an improvement in hearing by 20-30 dB.

Objective examination revealed a total perforation of the left tympanic membrane. A fistula with a skin defect (Figure 1) was observed in the retroauricular region, pathologically communicating with the mastoid cavity. The facial nerve was intact, and no nystagmus was detected. Audiotympanometry showed left-sided conductive hearing loss of 60-70 dB (Figure 2). A bacteriological examination of the ear swab was performed, and no pathogenic microorganism was identified.

**Figure 3:**
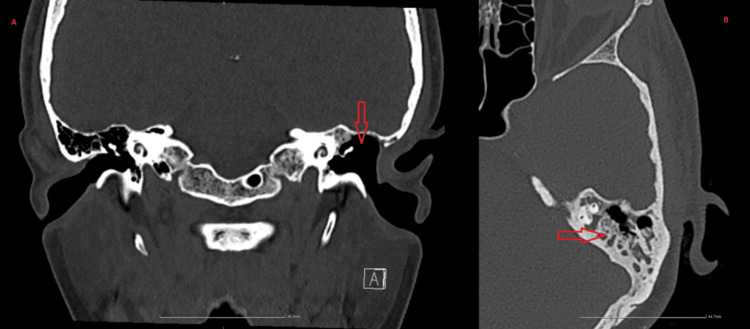
Computed tomography of the temporal bone: partially resectioned mastoid process A: Coronal view; B: axial view On computed tomography of the temporal bone, the following was noted: partial resection of the mastoid process, a viscous fluid accumulation dorsally in the mastoid air cells, and pneumatization of the tympanic cavity on the left side.

A computed tomography (CT) scan of the temporal bone was performed, revealing partial resection of the mastoid process. A viscous fluid accumulation was noted dorsally in the mastoid air cells (Figure 3). Pneumatization of the tympanic cavity was preserved on the left side. The auditory ossicles were distinguishable but slightly deformed. The diagnosis was established as chronic suppurative otitis media, chronic mastoiditis, and conductive hearing loss.

Based on subjective and objective findings, a surgical intervention was planned, including radical ear obliteration, canaloplasty, tympanoplasty, fistula closure, and functional hearing restoration.

Under general anesthesia, local infiltrative anesthesia was administered retroauricularly. An incision was made, extending to the mastoid apex, and the periosteum was dissected from the linea temporalis to the mastoid apex and from the spina suprameatum to the posterior mastoid border. The auricular cartilage was mobilized.

A radical mastoidectomy was performed, extending to the dura, sinodural angle, and semicircular canals. The posterior wall of the external auditory canal was removed. The bony canal of the facial nerve was lowered to the nerve level. A radical cavity was created and fully sanitized from the inflamed mucosa.

The oval window was covered with centrally perforated cartilage, forming a support (footplate shoe) for a hearing prosthesis. A total hearing prosthesis was inserted. The posterior wall of the external auditory canal was reconstructed using artificial bone powder and mobilized cartilage. A new tympanic membrane was created using cartilage and periosteum, which was placed over the total implant.

The mastoid cavity was obliterated with artificial bone impregnated with antibiotics. A skin flap was mobilized from the lateral surface of the neck and grafted into the external auditory canal. The retroauricular incision was closed in layers, and the fistula was sealed deeply using temporomastoid periosteum, with the skin sutured continuously. As postoperative measures, silicone sheets and antibiotic-soaked gel foam were placed in the external auditory canal, and a pressure dressing was applied to the ear.

The patient was hospitalized for two bed days, during which she received antibiotic therapy with ceftriaxone and ear dressings. The patient was discharged in satisfactory condition. At the infectious disease specialist's recommendation, oral antibiotic therapy with Orcipol (ciprofloxacin + ornidazole) was continued. The sutures were removed on the fourteenth day post-surgery, and detamping was performed on the tenth day.

**Figure 4:**
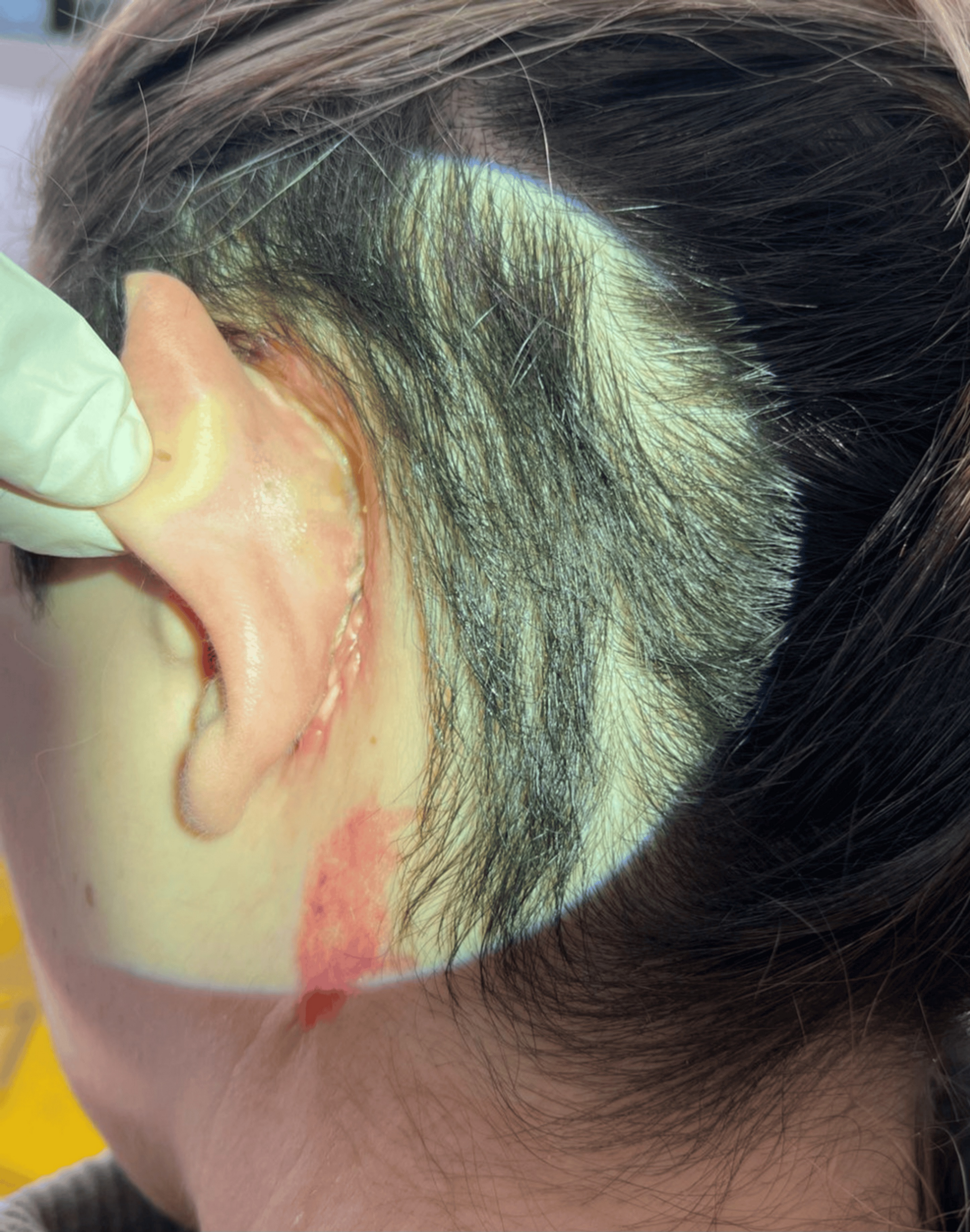
Image taken one month post-surgery, showing the closed cutaneous-mastoid fistula

One month after the surgery, hearing evaluation was performed through audiotympanometry, revealing an improvement in hearing by 20-30 dB (Figure 2). Objective assessment showed that the retroauricular fistula was closed (Figure 4), and the tympanic membrane was restored. The patient was scheduled for follow-up once a month.

## Discussion

From an epidemiological perspective, chronic mastoiditis can manifest in all age groups, with underlying factors including both genetic predisposition as well as impaired middle ear aeration and local infections [6-9]. In cases of chronic mastoiditis and tympanic membrane perforation, there are only two treatment alternatives: conservative, which aims to preserve a dry middle ear, and surgical, where the goal is both the removal of the infectious focus and the functional restoration of the ear [9,10].

Following several unsuccessful surgical interventions, the patient developed a retroauricular cutaneous-mastoid fistula, which served as a source of infection. Due to chronic mastoiditis and tympanic membrane perforation, the patient experienced hearing loss. Recurrent purulent otorrhea significantly impaired the patient’s quality of life, and her general condition was concerning. Based on the patient’s medical history, our multidisciplinary team decided to proceed with surgical intervention. The patient underwent a radical cavity obliteration, canaloplasty, and closure of the cutaneous-mastoid fistula. To prevent infection, both inpatient and outpatient antibiotic therapy were administered. With proper care and continuous monitoring by the otorhinolaryngologist, the patient and medical team achieved the desired outcome.

## Conclusions

This case demonstrates the successful surgical intervention performed to close a rare complication of mastoidectomy: the cutaneous-mastoid fistula. Through a multidisciplinary approach and careful planning of treatment tactics, the desired outcome was achieved. Following several unsuccessful surgeries, the patient’s hearing improved, radical cavity obliteration was performed, and the cutaneous-mastoid fistula was closed, resulting in an overall improvement in the patient’s quality of life.
